# Social consequences of mass quarantine during epidemics: a systematic review with implications for the COVID-19 response

**DOI:** 10.1093/jtm/taaa192

**Published:** 2020-10-13

**Authors:** Isaac Yen-Hao Chu, Prima Alam, Heidi J Larson, Leesa Lin

**Affiliations:** Department of Public Health, Environments and Society, Faculty of Public Health and Policy, London School of Hygiene and Tropical Medicine, London, UK; Department of Public Health, Environments and Society, Faculty of Public Health and Policy, London School of Hygiene and Tropical Medicine, London, UK; Department of Infectious Disease Epidemiology, Faculty of Epidemiology and Population Health, London School of Hygiene and Tropical Medicine, London, UK; Department of Health Metrics Sciences, University of Washington, Seattle, USA; Department of Infectious Disease Epidemiology, Faculty of Epidemiology and Population Health, London School of Hygiene and Tropical Medicine, London, UK

**Keywords:** Mass quarantine, lockdown, social consequences, COVID-19, epidemics, pandemics, psychological impact

## Abstract

Four billion people worldwide have experienced coronavirus disease 2019 (COVID-19) confinement. Such unprecedented extent of mobility restriction to curb the COVID-19 pandemic may have profound impacts on how individuals live, travel and retain well-being. This systematic review aims to identify (i) the social consequences of mass quarantine—community-wide movement restrictions—during previous and current infectious disease outbreaks and (ii) recommended strategies to mitigate the negative social implications of COVID-19 lockdowns. Considering social determinants of health, we conducted a systematic review by searching five databases (Ovid-MEDLINE, EMBASE, PsycINFO, China National Knowledge Infrastructure and the World Health Organization COVID-19 database) for publications from inception to 9 April 2020. No limitation was set on language, location or study type. Studies that (i) contained peer-reviewed original empirical evidence and (ii) focussed on non-epidemiological implications of mass quarantine were included. We thematically synthesized and reported data due to heterogeneous disease and country context. Of 3067 publications found, 15 original peer-reviewed articles were selected for full-text extraction. Psychological distress, heightened communication inequalities, food insecurity, economic challenges, diminished access to health care, alternative delivery of education and gender-based violence were identified as negative social consequences of community-based quarantine in six infectious disease epidemics, including the current COVID-19 pandemic. In contrast, altruistic attitudes were identified as a positive consequence during previous quarantines. Diverse psychological and social consequences of mass quarantine in previous and current epidemics were evident, but individual country policies had been highly varied in how well they addressed the needs of affected individuals, especially those who are socially marginalized. Policymakers should balance the pros and cons of movement restrictions, facilitate multisectoral action to tackle social inequalities, provide clear and coherent guidance to the public and undertake time-bound policy evaluations to mitigate the negative impact of COVID-19 lockdowns and to establish preparedness strategies for future epidemics.

## Introduction

The coronavirus disease 2019 (COVID-19) pandemic has become the largest global health emergency of the 21st century. On 30 January 2020, the World Health Organization (WHO) declared COVID-19 to be a Public Health Emergency of International Concern (PHEIC). By 30 August 2020, there were 24 854 140 reported infections and 838 924 deaths attributed to COVID-19 worldwide.^1^ While the world is pursuing curative treatments and vaccines, many governments have implemented community-wide movement restrictions—also known as ‘lockdown’ or ‘mass quarantine’—as interventions to stem the human-to-human transmission of COVID-19 by restricting individual mobility and face-to-face interaction.^2^^,^^3^ These restrictive measures ranged from working-from-home advisories and compulsory ‘shelter-in-place’ orders, to nationwide closure of schools, non-essential businesses and territorial borders.^4^ As of June 2020, an estimated 4.4 billion people have experienced COVID-19 confinement with border closures in >100 countries worldwide.^5^^,^^6^ Compared with the same period in 2019, the worldwide air passenger volume dropped by 63%, whereas road traffic flow reduced by half globally and plummeted 90% in 34 metropolises in April 2020.^6^^,^^7^ Such extent of mobility restrictions, travel bans and border closure to curb the COVID-19 pandemic have exceeded those in previous PHEICs over the last century.

As most activities in our society involve local, regional and international travel, movement restrictions attributed to mass quarantine may result in far-reaching social implications. Mass quarantine could be a double-edged sword: while community-wide containment has been shown to effectively decelerate the epidemic, it has profound impacts on how individuals live, travel and retain their well-being.^8^ Four review articles have identified negative implications of quarantine affecting public mental health and access to education.^9–12^ In the current response to the COVID-19 pandemic, scholars have raised concerns over travel, ethical, legal and equity challenges during confinement.^13–16^ Nevertheless, no studies have systematically assessed the social consequences of mass quarantine, defined as the impact of large-scale population-based containment with movement restrictions on individuals in specific social contexts.^17^ Little is known about the negative implications of community confinement that countries should consider in developing mitigation strategies in managing the current COVID-19 pandemic and preparedness for future epidemics. Therefore, we conducted a systematic review to identify: (i) the social consequences of mass quarantine during infectious disease outbreaks and (ii) recommended strategies to mitigate the negative social implications of COVID-19 movement restrictions.

## Methods

For the purpose of this study, we defined mass quarantine as measures that restricted physical contacts and mobility of either at least 10 000 people or all residents in specific jurisdictions (e.g. village, city and province). We searched publications on EMBASE, Ovid-MEDLINE and PsycINFO databases on 12 March 2020 and updated on 9 April 2020 when China ended the nationwide lockdown. Articles published from inception of the databases to 9 April 2020 were searched, with no limitation on language, location or study type. Our Boolean search strategy (Supplementary 1) combined terms related to mass quarantine (e.g. ‘quarantine’, ‘lockdown’ and ‘social distanc*’), diversified social consequences (e.g. ‘soci*’, ‘econom*’, ‘employ*’, ‘psych*’, ‘transport*’ and ‘educat*’) and infectious diseases (e.g. ‘SARS’, ‘COVID-19’, ‘coronavirus’, ‘MERS’, ‘Ebola’ and ‘H1N1’). Additionally, on 9 April 2020, we expanded the search to include the China National Knowledge Infrastructure and the WHO COVID-19 database. We manually scanned published review papers for relevant titles and contacted authors for clarifications and additional studies. We followed the PRISMA guidelines for design, analysis and interpretation of results. The protocol is registered with PROSPERO (CRD42020183756).

Two authors (I.Y.C. and P.A.) independently screened all searches by title and abstract. Documents referring to both social consequences and mass quarantine were considered eligible and then further reviewed in full text by I.Y.C. and P.A. We excluded studies that contained no peer-reviewed original empirical evidence (e.g. thesis, book chapters and reviews) or focussed only on epidemiological implications of mass quarantine (e.g. estimates of infection, rates/risks of diseases and reproduction numbers). Before data extraction, a coding framework (Supplementary 2) was developed centring on the concept of social determinants of health, defined as the environmental and social conditions in which people are situated (e.g. food, education and economic stability) that affect the health outcomes of human beings.^18^ The reported themes were driven by the data and revised through iterative discussions among three authors (I.Y.C., L.L. and P.A.). Two authors (I.Y.C. and P.A.) conducted data extraction independently and compared the results. Three authors (I.Y.C., L.L. and P.A.) discussed and agreed on the extraction of full-text articles. Considering quality assessment, the Mixed Methods Appraisal Tool (MMAT, version 2018)^19^ was applied to evaluate qualitative, quantitative and mixed-methods studies; ethics articles were examined using the five-item ethics critical appraisal matrix by Jansen and Ellerton.^20^ Studies satisfying at least four of five criteria were considered ‘high quality’. Two authors (I.Y.C. and P.A.) appraised all included studies in full-text and discussed quality assessment results with L.L. if no consensus was reached. The results of the quality appraisal were used to inform our evidence synthesis and further discussion. None of the eligible studies were excluded based on the results of our quality appraisal.

**Figure 1:**
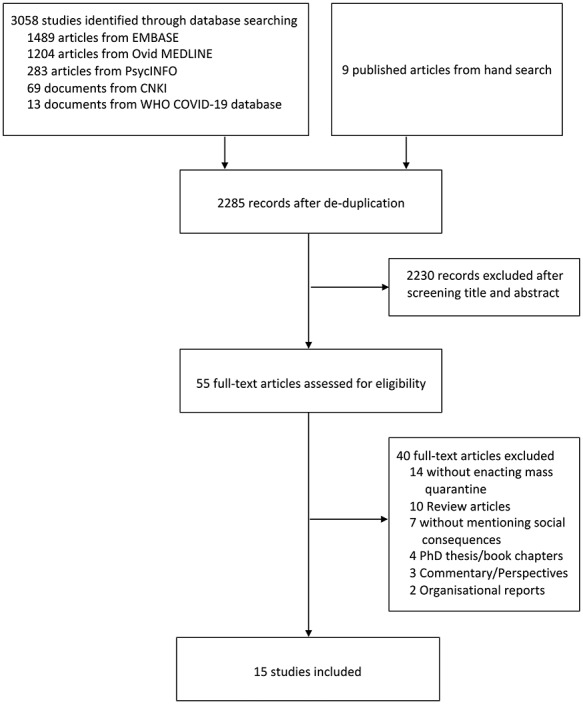
Process of study selection per the PRISMA statement

## Results

### Study characteristics

After screening the titles and abstracts of 3067 publications, we included 55 citations in the full-text assessment and extracted data from 15 eligible studies (Figure 1). Of all 15 included studies (Table 1), 8 focussed on the 2003 severe acute respiratory syndrome (SARS) outbreak in Canada, China and multi-country comparisons,^22^^,^^23^^,^^25–27^^,^^30^^,^^32^^,^^33^ followed by the 2014–16 Ebola virus disease (EVD) outbreaks in Sierra Leone and Liberia (*n* = 4),^21^^,^^29^^,^^31^^,^^34^ the 2020 COVID-19 pandemic (*n* = 2) in China and worldwide^28^^,^^35^ and the 2009/2010 influenza A (H1N1) pandemic in Canada (*n* = 1).^24^ The reported periods of quarantine varied from 7 days to 1 month. Table 2 presents details of quarantine measures and the context of infectious disease outbreaks.

**Table 1:** Characteristics of 15 included studies in the systematic review

Author	Country	Disease	Quarantine duration reported	Research design	Research measures	Study population	Primary outcomes	Theoretical approaches reported
Abramowitz *et al.* (2015)^21^	Liberia	EVD	21 days	Qualitative	Focus groups and interviews	386 community leaders	Optimal practices and innovative local strategies for EVD containment	Participatory rural appraisal models
Blendon *et al*. (2003)^22^	Canada	SARS	Not specified	Quantitative	Telephone surveys	501 Canadians who experienced mass quarantine	Knowledge, attitude of and precautionary measures against SARS	Not specified
Cava *et al*. (2005)^23^	Canada	SARS	9 days (Mean quarantine period)	Qualitative	Interviews	21 residents with contact history	Experience of home quarantine	Not specified
Charania and Tsuji (2013)^24^	Canada	H1N1	NA (thought experiment)	Qualitative community-based participatory	Interviews	Nine health care informants	Effectiveness and feasibility of implementing interventions to mitigate influenza pandemic in remote and isolated First Nations communities	Community*-*based participatory research
DiGiovanni *et al*. (2004)^25^	Canada	SARS	Up to 10 days	Mixed methods	Focus groups, interviews and telephone-based survey	35 residents for interview; 195 health care workers and 1509 residents for two respective surveys	Factors affecting compliance to quarantine	Not specified
Gostin *et al*. (2003)^26^	Multiple countries (Canada, China, Hong Kong, Singapore and Vietnam)	SARS	NA	Ethical analysis	Evidence synthesis	NA	Ethical and legal justifications on restrictions of privacy, liberty and movement in control of SARS outbreaks	Precautionary principle, least restrictive/intrusive alternative, justice and transparency
Hawryluck *et al*. (2004)^27^	Canada	SARS	Not specified	Quantitative	Web-based survey	129 respondents	Psychological effects of quarantine	The Impact of Event Scale-Revised and the Center for Epidemiologic Studies Depression Scale
John *et al*. (2020)^28^	Multiple countries	COVID-19	NA	Ethical analysis	Evidence synthesis	NA	Gender-based violence in previous and current public health emergencies	Not specified
Kodish *et al*. (2019)^29^	Sierra Leone	EVD	21 days	Qualitative	Interviews	42 informants for organizations and communities	Impact of EVD on nutrition sectors and factors for effective nutrition interventions in Sierra Leone	Not specified
Mihashi *et al*. (2009)^30^	China	SARS	Not specified	Quantitative	Survey	187 respondents comprising printing company workers, university faculty members and their families and non-medical students	Predictors of psychological disorders after SARS outbreaks	An assistance model previously developed by the authors for the 1988 dysentery outbreak in the USA
Pellecchia *et al.* (2015)^31^	Liberia	EVD	21 days	Qualitative	Focus groups and interviews	462 residents of neighbourhoods diagnosed with EVD	Social implications of EVD containment with regard to communities’ perception of and response to restrictive measures	Not specified
Reynolds *et al.* (2008)^32^	Canada	SARS	From 7·8 to 8·7 days (Median from sub-group analysis)	Quantitative	Mailed survey	1057 respondents	Psychological impact of quarantine (feelings, fears of developing SARS, stigmatization and symptoms of post-traumatic stress disorder)	The Impact of Event Scale-Revised
Tracy *et al.* (2009)^33^	Canada	SARS	Not specified (assessing public attitudes toward quarantines)	Quantitative	Computer-assisted telephone interviews	500 residents of Toronto and Regional Municipality of York	Perceptions of quarantine (justifications, sanctions, burdens and safeguards)	Harm Principle, Least Restrictive Means, Reciprocity Principle, and Transparency Principle
Wilken *et al.* (2017)^34^	Liberia	EVD	21 days	Qualitative	Interviews	115 village residents	Knowledge, attitude and practices of EVD control	Not specified
Zhang *et al.* (2020)^35^	China	COVID-19	One month into the lockdown of Wuhan, China	Quantitative	Cross-sectional survey	369 adults not epidemiologically affected by COVID-19	Mental health conditions and life satisfaction	The 12-item Short Form Physical and Mental Health Summary Scales, the Six-item Kessler Psychological Distress Scale and the Satisfaction with Life Scale

NA: not applicable.

**Table 2:** The details of quarantine measures among 13 of 15 selected studies^a^

Disease	Country	Income of economies	Year	Area affected	Number of populations affected	Quarantine measures for individuals	Type of enactment	Study included in the Review
COVID-19	China	UMIC	2020	Region	57 million people in Hubei Province^35^	No public transportation Restricted movement as one household lead can leave home on alternative days with temperature monitoring at checkpoints Fourteen-day quarantine after travel	Compulsory with administrative orders (police enforcement if necessary)	Zhang *et al.* (2020)^35^
H1N1 Influenza	Canada	HIC	2009	Country	33 509 people diagnosed in Canada^a^	Voluntarily stay at home and avoid mass gathering^a^	Advisory	Charania and Tsuji (2013)^24^
EVD	Liberia	LIC	2014–2016	Region	Approximately 75 000 living in West Point, Monrovia^a^	Home-based quarantine of villagers with strict 21-day movement ban Daily active temperature monitoring	Compulsory with law enforcement and military force	Abramowitz *et al.* (2015)^21^ Pellecchia *et al.* (2015)^31^ Wilken *et al.* (2017)^34^
EVD	Sierra Leone	LIC	2014–2016	Country	Estimated 4.5 million^a^	A 3-day national lockdown and 21-day lockdown in high epidemic areas Schools and public places closed Curfew enacted	Compulsory with law enforcement (jail sentence) and military force	Kodish *et al.* (2013)^29^
SARS	Canada	HIC	2003	Region	25 000^a^	Home-based quarantine of close contacts of SARS patients for an average of 10 days The quarantine criteria were periodically reviewed by Toronto health officials	Advisory personal and household quarantine with maximum fines of $5000 Canadian dollars for violators	Blendon *et al.* (2003)^22^ Cava *et al.* (2005)^23^ DiGiovanni *et al.* (2004)^25^ Hawryluck *et al.* (2004)^27^ Reynolds *et al.* (2008)^32^ Tracy *et al.* (2009)^33^
SARS	China	UMIC	2003	City	30178^a^	Close contacts were quarantined for 14 days Home-based quarantine but allowed pre-approved movement School closure and restricted travel citywide	Compulsory with administrative orders (police enforcement if necessary)	Mihashi *et al.* (2009)^30^

Two studies (Gostin *et al*.^26^ and John *et al*.^28^) based on ethical scenarios are not listed.

^a^See Supplementary 3 for references.

LIC: low-income country, UMIC: upper middle income country, HIC: high-income country.

### Quality assessment

Thirteen empirical studies and two ethics papers were assessed using the MMAT and the ethical appraisal matrix, respectively (Table 3). Of all 15 included studies, 6 were of low quality and 9 (2 quantitative studies,^32^^,^^34^ all 5 qualitative studies^21^^,^^23^^,^^24^^,^^29^^,^^31^ and both ethics studies^26^^,^^28^) were regarded as high quality. Neither of the two mixed-methods studies^25^^,^^27^ on SARS in Canada was viewed as high quality because the rationale and integration of multiple methods were not reported. All instruments for measuring social consequences of mass quarantine were employed on an *ad hoc* basis, except that two studies of SARS applied the Impact of Event Scale-Revised.^27^^,^^32^

**Table 3:** Results of the quality assessment (*n* = 15) using MMAT^19^ and the ethics framework by Jansen and Ellerton^20^

Quantitative studies						
First author	Relevant sampling strategy to address research question	Representative sample of target population	Appropriate measurements	Low risk of non-response bias (≥80% response rate)	Appropriate statistical analysis to answer research question	High quality^a^
Blendon *et al.* (2003)^22^	✓	✓		✓		No
Mihashi *et al*. (2009)^30^	✓				✓	No
Reynolds *et al.* (2008)^32^	✓	✓	✓		✓	Yes
Tracy *et al.* (2009)^33^	✓		✓		✓	No
Wilken *et al.* (2017)^34^	✓	✓		✓	✓	Yes
Zhang *et al*. (2020)^35^	✓					No
Qualitative studies						
First author	Appropriate approach to answer research question	Adequate data collection methods to address research question	Adequate findings derived from data	Interpretation of results sufficiently substantiated by data	Coherence between data sources, collection, analysis and interpretation	High quality^a^
Abramowitz *et al.* (2015)^21^	✓	✓	✓	✓	✓	Yes
Cava (2005)^23^	✓	✓	✓	✓	✓	Yes
Charania and Tsuji (2013)^24^	✓	✓	✓	✓	✓	Yes
Kodish *et al.* (2019)^29^	✓	✓	✓	✓	✓	Yes
Pellecchia *et al.* (2015)^31^	✓	✓	✓	✓	✓	Yes
Mixed-methods studies						
First author	Adequate rationale for using mixed-methods design	Effective integration of different components of study	Adequate interpretation of outputs of qualitative and quantitative components	Divergences/ inconsistencies between quantitative and qualitative results adequately addressed	Components of study adhere to specific tradition quality criteria	High quality^a^
DiGiovanni *et al*. (2004)^25^			✓			No
Hawryluck *et al*. (2004)^27^						No
Ethics studies						
First author	Different points conflated and adequately addressed	Key term well defined with reasonable definitions	Premises are supported with evidence followed by logical conclusions	All relevant counterarguments are addressed	Arguments or explorations of issue relevant to target practices	High quality^a^
Gostin *et al*. (2003)^26^	✓	✓	✓	✓	✓	Yes
John *et al*. (2020)^28^	✓	✓	✓		✓	Yes

^a^Studies satisfying at least 80% (four of five) assessment criteria are considered as high quality.

### Social consequences of mass quarantine

We identified seven types of social consequences of mass quarantine (Table 4): psychological distress (*n* = 11), heightened communication inequalities (*n* = 9), food insecurity (*n* = 8), economic challenges (*n* = 7), diminished access to health care (*n* = 6), disruptive education (*n* = 4) and gender inequity and violence (*n* = 3).

**Table 4:** Synthesized results and recommendations on mitigating the social consequences of quarantine

Consequences identified from 15 included studies	Themes	Examples	Recommendations from 15 included studies
Psychological and mental distress	Emotional conditions	Annoyance, anxiety, boredom, disappointment, fear of infection, isolation, loneliness and mistrust	Provide both personal consultations and community psychological support to vulnerable populations
	Symptoms of mental disorders	Post-traumatic stress disorder and depressive disorders	
	Stigma and discrimination	Self-isolated individuals and EVD survivors were regarded as EVD spreaders; anti-Asian racism during the SARS outbreak in Canada	
Heightened communication inequalities	Public distrust of governments’ responses	Growing distrust of governments’ compulsory lockdown of slums in Liberia’s EVD outbreak	Provide comprehensive support and transparent information on quarantine Combating misinformation by adapting context-specific approaches and supporting research efforts Prevent implementation failure by engaging with socially vulnerable populations
	Misinformation on quarantine measures	Contradictory quarantine instructions from public health officials, mass media and unauthorized analysts during Canada’s SARS outbreak	
	Limited compliance to quarantine orders with increased risks of health	Overcrowding, poverty and lack of health care were reported as determinants of individuals’ compliance to quarantine in EVD, H1N1 and SARS outbreaks	
Food insecurity	Food production and transportation	Little grain harvesting during EVD confinement in Sierra Leone; delayed food transportation due to travel restrictions for SARS containment in China	Nutritional preparedness, such as food production, access, distribution and monitoring should be planned and timely implemented
	Food access and storage	Reduced access to food during mass quarantine against SARS in China and Canada as well as EVD in Sierra Leone and Liberia	
Economic challenges	Interrupted international industries	Agricultural production, leisure business and tourism at domestic and international levels during EVD and SARS outbreaks	Provide equitable financial compensation (e.g. universal credits or extensions of business relief) to ensure the financial security of those under quarantine
	Closure of local business entities	Shutdown of non-essential business and reduced business revenue due to decreased demands with existing costs of employment in H1N1 preparedness plans	
	Reduced personal incomes	Unemployment and unstable incomes for part-time or are self-employed individuals during Canada’s SARS outbreak	
Diminished access to health care	Access to essential medicine and services for noncommunicable diseases	Increased number of deaths and complications from preventable health conditions during EVD outbreak in Liberia; lacked access to regular prescriptions in Canada’s SARS outbreak and China’s COVID-19 outbreak	Strengthen capacity of health care systems and equitable health care access
	Reduced health-seeking behaviour	Drop-outs of nutrition screening and hiding treatable illnesses during EVD outbreaks in Sierra Leone and Liberia	
Disruptive of education	Remote and online education	Web-based learning resources for adolescents and students during SARS outbreak in Canada	Ensure resource allocation for education innovation and platforms
	Caregivers as educators	Community members took responsibility for children’s education during Liberia’s EVD outbreak and Canada’s H1N1 outbreak	
Gender inequity and violence	Gendered home care responsibility	Women’s default role as caregivers at home during the EVD outbreak in Liberia	Establish gender-inclusive norms in national policymaking and global health governance
	Gender-based violence	Increased numbers of women experiencing domestic violence in China and the UK during the COVID-19 pandemic	

### Psychological distress

Eleven articles from various geographical and disease contexts highlighted the psychological implications of mass quarantine as emotional distress and symptoms of mental illness.^22–25^^,^^27–32^^,^^35^ Among people in or after quarantine, some experienced emotional distress, including: annoyance,^32^ anxiety,^25^^,^^29^ boredom,^23^^,^^25^^,^^32^ disappointment and life dissatisfaction,^24^^,^^32^^,^^35^ fear of infection,^23^^,^^25^^,^^30^^,^^34^ isolation,^23^^,^^25^^,^^32^ loneliness^25^^,^^32^ and mistrust.^31^ Mobility restrictions could disproportionately impact unemployed individuals; for example, in Zhang *et al*.’s^35^ study of the well-being of individuals during the COVID-19 lockdown, there was strong evidence that those who stopped working had poorer mental health conditions than those still employed [a decrease of 2.60 points in Mental Composite Scale; 95% confidence interval (CI) = −0.05 to −5.16]. While one study revealed that people under physical distancing and movement restrictions suffered insomnia and depression,^30^ Hawryluck *et al*.^27^ reported that around one-third of respondents suffered from symptoms of post-traumatic stress disorder and depression (28.9% and 31.2%, respectively). Nevertheless, this result was prone to reporting bias as the survey response rate was <1%^27^; confirmation of psychiatric disorders required further clinical diagnosis, on which data were not available.

In contrast, altruistic attitudes during mass quarantine were reported in three studies. The majority of respondents in a Canadian study agreed that following quarantine orders would protect others from contracting SARS.^33^ Interviewees mentioned how community members offered emotional support and took care of orphans during the mass quarantine in the Toronto SARS epidemic and in the Liberia EVD outbreak, respectively.^23^^,^^29^

Stigma and labelling may pose further psychological challenges to people under quarantine. Pellecchia *et al*.^31^ pointed out that the state-enforced quarantine in Liberia during the 2014–16 EVD outbreak heightened the extent of stigma experienced by residents under compulsory isolation with travel ban regardless of Ebola virus infection. Those who self-isolated were treated as disease spreaders, and their behaviour was morally judged by other community members; a religious leader worried that misinformation during mass quarantine heightened the mistrust between racial and religious groups, as some interviewees accused ethnic minorities of spreading diseases. A Canadian study noted that around one-fifth of survey respondents avoided going to businesses or meeting people with a potential travel history to Asia during the SARS outbreak in Toronto.^22^ In addition to the impact on mental well-being, stigma derived from mass quarantine may inhibit affected individuals from accessing food and other essential items. One study mentioned that survivors of EVD in Sierra Leone experienced rejection from food sellers.^29^

### Heightened communication inequalities

Nine studies stressed how mass quarantine aggravated inequalities in individuals’ access to, understanding of, and actions on prevention and control of infections.^21^^,^^23–26^^,^^31–34^ A Canadian study found that racial and linguistic minorities might suffer a higher risk of having inaccurate information on measures of SARS confinement,^23^ which might result from inadequate literacy of the audience or a lack of clarity of the messages. Another study of the EVD outbreak in Liberia underlined that slum dwellers distrusted the government due to a lack of information on military-enforced lockdown targeting their residence.^31^ In addition, identifying trustworthy information became challenging for the public due to diverse and unverified sources as well as heightened uncertainty during disease outbreaks.^23^^,^^25^^,^^27^ Participants from three studies of the SARS outbreak in Toronto stressed that authorities did not provide clear and consistent messages on why, how and, how long to enact quarantine, and that they were unable to contact designated public health staff.^23^^,^^25^^,^^27^ Moreover, contradictory quarantine instructions from public health officials, mass media and expert opinions widened communication inequalities, made it difficult for interviewees to comply with quarantine orders and drove the public to take on word-of-mouth recommendations that might or might not be true.^25^ Two studies of SARS confinement reported that being health care workers was predictive of correct knowledge of quarantine and that female and older (>65 years old) respondents were more likely to accept the use of mass quarantine.^32^^,^^33^ Housing conditions, poverty and the presence of health care facilities were reported as determinants of individuals’ compliance with mass quarantine.^21^^,^^24^^,^^31^ Gostin *et al*.^26^ argued that people in poverty could not afford space for physical distancing in their households during SARS outbreaks; similar challenges were found among the First Nations population in subarctic Canada.^24^ Liberian community leaders argued that, despite being aware of EVD, they could not respond effectively to EVD control without a functioning health care system.^21^ Another Liberian study of EVD suggested that high quarantine compliance among village residents was attributed to designated health care personnel on-site.^34^

When it comes to recommendation on communications, Abramowitz *et al*.^21^ suggested developing community-based peer education programmes and improving communication infrastructure to reduce the negative social impacts of quarantine. Gostin *et al*.^26^ stressed that governments should improve the transparency of decision-making on community-wide movement restrictions and inform the public about how outbreak surveillance works to avert unnecessary panic. Pellecchia *et al*.^31^ stated that top-down enforced lockdowns without community engagement may fuel distrust of authorities and resistance to restrictive measures, which could result in ineffective outbreak control.

### Food insecurity

Mass quarantine-induced mobility restrictions impacted every step of the food supply chain, including production,^29^ transportation,^24^^,^^29^^,^^30^ access^22–24^^,^^27^^,^^29^^,^^31^^,^^34^ and storage.^23^^,^^29^ One study identified that, because the quarantine period overlapped with the harvest season, workers and farmers could not travel to their filed for harvesting agricultural products, which then created downstream effects on the food system.^29^ Indigenous people in Canada advocated against full border closures to retain the supply of basic needs, whereas Mihashi *et al*.^30^ argued that delayed supply caused by limited transportation could aggravate psychological distress (e.g. anxiety) among Chinese individuals.^24^ Pellecchia *et al*.^31^ revealed that some villagers in Liberia disobeyed the enforced quarantine order due to intermittent food supply. Regarding food storage, interviewees in Sierra Leone worried about their own food stock,^29^ whereas some Canadians were concerned about how others in economic difficulties preserved food under movement restrictions.^23^ Food access during quarantine varied and could be country specific. Levels of food inaccessibility were reported at 4% (*n* = 501) in one study of the SARS outbreak in Canada^22^ and at 50% (*n* = 16) in another study of the EVD outbreak in Liberia.^34^ Liberians stressed that the compulsory quarantine order damaged the tradition of mutual support between village dwellers and left those self-isolated unaided and starving.^31^ When asked about ways to increase food security, the majority of surveyed respondents in two studies agreed that governments should provide quarantined individuals with food, shelter and other basic needs.^33^^,^^34^

### Economic challenges

Mass quarantine had widespread economic impacts at both business and individual levels by limiting personal movement and transportation of goods. Three studies reported that travel bans during mass quarantine might impact agricultural production, leisure business and tourism.^22^^,^^26^^,^^29^ Kodish *et al*.^29^ explored the impact of the EVD outbreak on the food supply chain in Sierra Leone. They indicated that decreased production of grains and reduced mobility of traders interrupted the domestic and international flow of agriculture trade. In a study of Canadians’ responses to the 2003 SARS outbreak, 22% of surveyed respondents (*n* = 355) in Ontario closed restaurants and cancelled social activities.^22^ The authors argued that these precautions, intersecting with SARS-related stigma against Asian businesses, could potentially harm the local economy. Gostin *et al*.^26^ expressed that travel restrictions caused enormous damage to businesses relying on mobility and individuals having economic interests in tourism. None of the studies provided macroeconomic data (e.g. change in gross domestic product per capita) to further support their findings.

When considering the economic impact on individuals, reduced personal incomes, unemployment and concerns about additional costs of employment were identified as the consequences of community-wide containment. In two studies of the SARS outbreak in Toronto, Canada, 10.0–25.7% of surveyed respondents experienced reduced or no payment due to missing work.^22^^,^^32^ Loss of income following unemployment was the key consequence raised in qualitative research on the SARS and EVD outbreak. DiGiovanni *et al*.’s^25^ study argued that individuals who work part-time or are self-employed had no guarantee to an income, as local governments delayed offering financial compensation to asymptomatic people under quarantine who could not work without travel. A qualitative study reported that residents in Sierra Leone during the EVD outbreak were unable to work due to village-based quarantine, which further impacted labour force supply and the agricultural cycle.^29^ Their results highlighted the interconnectivity between the business economy and employment. Regarding recommendations on balancing implications between business economics and employment, interviewees from the First Nations population in one Canadian study suggested closing ‘non-essential community workplaces’ to reduce the expenses for compensating quarantine employees.^24^ Another Canadian study showed that, while 88% of respondents agreed that people should follow quarantine orders regardless of employment status, 68% argued that governments should compensate individuals for their lost earnings during quarantine.^33^

### Diminished access to health care

Mass quarantine affected health care access through the reallocation of health care resources to the outbreak emergency and by deepening health inequity among vulnerable populations. In one study of the EVD outbreak in Liberia, some informants observed an increased number of deaths and complications from preventable health conditions, as most medical facilities within reasonable travel distances were closed.^21^ The extent of health access varies by context. Two studies noted that some people under quarantine in the SARS and COVID-19 epidemics lacked access to regular prescriptions and health care services.^22^^,^^28^ Conversely, compared with pre-EVD situations, all villagers (*n* = 9) with sick family members in a study of the EVD outbreak in Liberia reported full access to medical care, which was supported by local governments’ medical transportation services.^34^

Three studies revealed changes in health-seeking behaviour during lockdowns, including reduced visits to nutrition screening, hiding treatable illnesses and seeking help from unverified sources.^21^^,^^29^^,^^31^ Kodish *et al*.^29^ underlined a drastic shift in public health priorities, as EVD management entirely replaced existing nutrition screening programmes at the beginning of the outbreak. They underscored that the reported reduction in malnutrition screenings resulted from movement restrictions under quarantine, behaviour changes in service users due to lack of trust and resource competition between the EVD outbreak management and humanitarian nutrition programmes, both of which were vital to survival.

### Disruptive education

Four studies stressed how school closures under lockdowns affected children and adolescents.^21^^,^^24^^,^^25^^,^^29^ During the EVD outbreak, informants in Sierra Leone reported no schooling in general, whereas several community members in Liberia took on the responsibility to educate children who had lost their parents.^21^^,^^29^ Two studies from both remote and urban areas in Canada reported coping strategies among affected caregivers and teenagers.^24^^,^^25^ In a study of H1N1 preparedness among indigenous citizens, some interviewees argued that adults with school-aged children could provide home schooling if both schools and workplaces were shut down in mass quarantine.^24^ In another study by DiGiovanni *et al*.,^25^ adolescent respondents contended that they could obey quarantine orders and learn from home given that mobile connections and web-based learning platforms were available.

### Gender inequality and violence

Three articles highlighted how mass quarantine and movement restrictions could deepen gender inequality and gender-based violence.^21^^,^^28^^,^^30^ Abramowitz *et al*.^21^ described the inequality of housework distribution, as women were regarded as default caregivers of children and sick family members at home. Female participants described their strategies for making protective equipment using available but non-standardized materials to deliver home-based care during the EVD outbreak. The authors also argued that women might delay visits to hospitals because being hospitalized would risk their family care responsibilities. John *et al*.^28^ maintained that the trend of domestic violence cases escalated since the COVID-19 lockdown in both Hubei province, China and the UK. They emphasized the invisibility of gendered home care in countries undergoing mass quarantine and called for rights-based support to prevent violence against women. Considering the implications of quarantine on men, one Chinese study argued that male respondents under quarantine were 3.5 times more likely (odds ratio = 3.5, 95% CI = 1.6–7.7) to report psychological disorders (defined as scoring seven and more in the 30-item General Health Questionnaire), albeit without psychiatric diagnoses for clinical confirmation.^30^

## Discussion

Our review identified seven negative social consequences of community-wide movement restrictions, including negative impacts on mental well-being, communication, food security, economy, health care access, education and gender equality. Altruism was the only recognized positive consequence. In countries where mass quarantine was introduced in response to epidemics, the needs of populations affected by movement restrictions, especially those who are socially and economically vulnerable, were not sufficiently addressed.

Consistent with previous studies,^10^^,^^36^^,^^37^ our review presents a range of negative psychological impacts of mass quarantine, such as low life satisfaction, emotional isolation and fear of infection.^23–25^^,^^30^^,^^32^^,^^34^^,^^35^ These impacts may converge with other determinants of health and further exacerbate negative societal effects of mass quarantine on individuals. As the period and scale of travel restrictions attributed to COVID-19 lockdowns have largely exceeded those of previous outbreaks, long-term psychological implications may disproportionately affect populations suffering from economic hardship, such as those unemployed, unable to work from home or experiencing business closures.^38^^,^^39^ Moreover, limited access to health care services overloaded by COVID-19 pandemics may exacerbate such psychological implications.^40^ Physical distancing and mobility restrictions challenge the service delivery of face-to-face consultations to individuals with pre-existing conditions, whereas issues like substance abuse can be left undealt (not only) in countries with little preparedness in mental health services.^41^^,^^42^

In contrast to negative implications, altruism was identified as a positive consequence from research on the EVD and SARS outbreaks.^23^^,^^29^^,^^33^ COVID-19 offers an opportunity for comprehensive investigations on other positive consequences of mass quarantine. While promoting altruism, policymakers should develop culturally competent and context-specific interventions,^43^^,^^44^ facilitate the use of technology to retain social connections and increase the capacity of health care services with digital innovations.^45^

Our results highlight the unintended and negative impacts of mass quarantine, including reinforcing stigma against social minorities,^31^ aggravating misinformation^31^^,^^46^ and undermining public trust in governments.^29^^,^^31^ During COVID-19 lockdowns, these consequences have added to the emotional burden and heightened existing communication inequalities in society, defined as uneven abilities of individuals or social groups in accessing, processing and disseminating information on health topics.^47^ Such inequalities comprise discrimination against Asian populations,^48^ unverified claims about lifting quarantine measures^49^ and breaking quarantine orders by organizing unauthorized gatherings and anti-quarantine protests.^50^ Often, communication inequalities are compounded by poor information governance across authorities during outbreaks, such as non-justified decision-making, inconsistent instructions and non-synchronized implementation of mass quarantine.^23^^,^^25^^,^^27^^,^^51^ Previous studies have suggested negative associations between the extent of communication inequality and countries’ outbreak preparedness,^49^^,^^52^ hindering the effectiveness of containment efforts and weakening societies’ capability in response to health emergencies.^53–55^ Further, communication inequalities need to be addressed together with emotional, social and political determinants of health in policy intervention,^56^ the last of which refers to the effects of power, institutions and ideologies on population health at various levels and culture of political systems.^57^ Policymakers can ensure communication equality by designing equity-based communication messages (e.g. information tailored to individual needs by age, education level and language use) based on correct data and risk-adapted measures, consulting social minority representatives in developing supplementary measures to lockdowns, learning from countries successfully tackling COVID-19 misinformation (in mass media or by politicians) and supporting research efforts.^53–55^^,^^58^ Transparency, community engagement and context-tailored strategies for combating misinformation are key to mitigating communication inequalities.^53^^,^^54^^,^^59^

Our review also identified a research gap in the gender impacts of mass quarantine. With emerging evidence on how COVID-19-induced mobility restrictions have disproportionally impacted women (e.g. gender-based violence),^60^^,^^61^ sexual minorities^62^^,^^63^ and ethnic minority groups worldwide,^64^^,^^65^ COVID-19 responses should leverage efforts to mitigate, rather than heighten, social disparities among gender and racial minorities. Further research on the needs of vulnerable populations during confinement with appropriate considerations based on verified data is crucial to informing equitable and sustainable interventions.

We call for attention to the contextual factors of policy interventions in the current and post-COVID-19 period. Evidence showed that negative social implications of lockdowns may heavily burden countries unprepared for public health emergencies. Food insecurity refers to hoarding supplies and panic buying in high-income countries, but it may result in starvation and famine at population levels in low-income countries. Distance learning may seem feasible in urban settings but highlights the digital inequality in remote areas with limited network infrastructure as well as in people living in poverty.^66^ As the breadth and depth of social consequences differ in various contexts, a one-size-fits-all policy balancing epidemiological and social impacts of mass quarantine does not exist. Interventions need to address pre-existing inequalities as well as those heightened by COVID-19 lockdowns. In other words, individual vulnerabilities, bureaucratic barriers and cultural competency of governments may determine whether a policy will alleviate or exacerbate the societal implications of mass quarantine. For instance, reports have shown that the Paycheck Protection Program (PPP)^67^ in the USA may not effectively aid business entities and individuals in states severely affected by COVID-19 as most lenders (i.e. banks) select borrowers based on existing relationships.^68^^,^^69^ Refugees and immigrants were reported to have no access to social relief package in the ongoing COVID-19 lockdown in South Africa.^70^ To maximize positive impact and minimize unintended consequences of policy interventions, policymakers should consider existing social inequalities, equity-based implementation processes and mechanisms of action before devising their revival plans, even before issuing any lockdown order.^71^ Clear guidelines, expanded testing and rights-based use of technology for contact tracing may facilitate sustainable policies and avoid prolonged mobility restrictions due to COVID-19.^72^^,^^73^^,^^74^ Future studies should continue to explore how, and to what extent, political determinants of health affect the social consequences of COVID-19 confinement across various socio-economic and cultural contexts.

Several limitations bear mentioning in our study. Firstly, the scarcity of COVID-19-related studies at the time of our database searching (i.e. April 2020) has limited the transferability of our results as the amount of research on COVID-19-related movement restrictions has exponentially grown since then and data on the longer-term social consequences of COVID-19 mass quarantine will not be available until much later. The majority of the included studies focus on SARS and EVD, whereas the scale and geographic locations of community-wide movement restrictions may not be comparable to the COVID-19 pandemic. Our results require careful interpretation, especially quantitative findings in studies without high-quality methodologies. Nevertheless, the consequences identified in our study contribute to knowledge by summarizing confirmed as well as plausible associations for future exploration among a myriad of COVID-19 publications. Secondly, most of the included studies are subject to sampling bias, with heterogeneous characteristics of research populations (e.g. urban, rural, high-income and low- and middle-income settings). Two studies were based on ethical scenarios rather than lived experiences of people in quarantine.^26^^,^^28^ Our findings may be generalized to neither all countries worldwide nor identity-specific individuals. While rapidly responding to research inquiries into COVID-19 confinement, future research should aim to minimize potential biases and consider the interaction among each of social determinants of health in countries with different political systems. Comparative studies assessing how sociopolitical factors influence the adoption, delivery and outcomes of financial assistance policies (e.g. PPP in the USA^67^ and Job Retention Scheme in the UK^75^) will contribute to implementation science in the post-COVID-19 era. Often, policymakers’ perceived priorities may determine the resources allocated for implementation, which result in varied effectiveness and (un)intended social consequences. For instance, the Swedish government implements voluntary quarantine to maintain business operations in the COVID-19 pandemic, despite researchers’ disputes and concerns over asymptomatic transmission.^76^ Reports showed that Sweden’s strategy did not result in economic growth but rather increased unemployment rates and exclusion from Nordic travel zone.^77^^,^^78^ Investigation on the ‘ripple effects’^79^^,^^80^ of policies at domestic and international levels will inform policymaking with better supplementary measures. Thirdly, none of the included studies provided macroeconomic estimates to reflect on the broader economic consequences of mass quarantine. The International Monetary Fund has forecast a worldwide recession caused by COVID-19 lockdowns.^81^ Our review cannot provide insights into the debate over cost-effectiveness of mass quarantine but offer evidence through the lens of individuals’ perceptions of economic hardship.^82^^,^^83^ More economic research is crucial to elucidating the profound economic effects of long-term lockdowns. Lastly, our findings did not thoroughly identify all the social consequences of mass quarantine. Issues such as climate change should not be neglected in the commitment to the UN Sustainable Development Goals by 2030, which all countries must reaffirm with global collaboration in the current and post-COVID-19 period. Recent reports suggested positive health effects of reduced air pollution during COVID-19 lockdown.^84^^,^^85^ Mass quarantine provides researchers with a window of opportunity for stressing the health gains of climate action on low carbon travel, investigating diverse and long-term biopsychosocial^86^ repercussions of movement restrictions with constructive suggestions^87–90^ and establishing frameworks to reduce social inequalities in the post-COVID-19 Anthropocene.^91^^,^^92^

## Conclusions

Mass quarantines can lead to multidimensional social consequences and may potentially heighten existing disparities across various contexts. Reducing social inequalities in every context needs to become a priority for countries to build resilience during the COVID-19 pandemic and to strengthen the preparedness for future emergencies. Whenever movement restrictions remain necessary in the current and post-COVID-19 period, policymakers should enact equity-based and context-specific interventions to mitigate socio-economic implications and mental health repercussions. In preparing to phase out restrictive measures, countries should facilitate multisectoral actions to tackle social inequalities, provide clear and coherent guidance to the public and undertake time-bound policy evaluations. Such efforts will minimize the negative consequences of the COVID-19 confinement and establish preparedness for future public health emergencies.

### PROSPERO registration

CRD42020183756.

## Ethical approval

Not required.

## Contributors

L.L. conceptualized the paper and developed the search and coding strategies together with I.Y.C. and P.A. I.Y.C. and P.A. searched, screened and assessed the quality of peer-reviewed articles advised by L.L. and H.L. I.Y.C. reviewed all selected publications and policy documents, extracted the data and wrote the first draft of the manuscript. I.Y.C. and L.L. discussed analysis and synthesis of policy documents. I.Y.C. and P.A. discussed discrepancies of quality assessment with L.L. I.Y.C. drafted the initial version of the manuscript. H.L., L.L. and P.A. revised the manuscript with inputs on policy recommendations. The final version of the manuscript was contributed to and approved by all authors. The corresponding author has full access to all the data in the study.

## Conflict of interest

None declared. The research activities, findings and conclusions expressed by authors contributing to this systematic review neither represent the view of nor involve any institution.

## Funding

This project is funded by Wellcome Trust (Grant no. 215373/Z/19/Z). I.Y.C. is funded by the Taipei Veterans General Hospital-National Yang-Ming University Excellent Physician Scientists Cultivation Programme (Scholarship no. S103-F-052) for his Doctor of Public Health research degree at LSHTM. P.A. is funded by the Economic and Social Research Council’s 1+3 Studentship for her PhD research degree at LSHTM. H.L. received grants from GSK and Merck to conduct research on vaccine acceptance.

## Supplementary Material

Supplementary_Material_160920_IC_taaa192
