# Supplementary material for: Social consequences of mass quarantine during epidemics: a systematic review with implications for the COVID-19 response
Source: J Travel Med. 2020 Oct 13;27(7):taaa192. doi: 10.1093/jtm/taaa192 (PMC7649384; doi:10.1093/jtm/taaa192)
Supplement: Supplementary_Material_160920_IC_taaa192 [file supplementary_material_160920_ic_taaa192.docx]

**Supplementary Material**

**Manuscript title:** Social consequences of mass quarantine during epidemics: a systematic review with implications for the COVID-19 response

**Authors:** Isaac Yenhao Chu, Prima Alam, Heidi Larson, Leesa Lin

**Supplement 1. Search strategies**

**Embase, Ovid-MEDLINE, and PsycInfo:**

1. (quarantine or lockdown or contain* or confine* or social distanc*).mp.

2. exp quarantine/ or exp patient isolation/ or exp Social Distance/

3. 1 or 2

4. ("social consequen*" or implication* or consequen*).mp.

5. soci*.mp.

6. econom*.mp.

7. (safe* or secur*).mp.

8. (psych* or mental*).mp.

9. trust*.mp.

10. (travel* or transport*).mp.

11. (educat* or campus or school or college or universit*).mp.

12. (job* or employ* or work).mp.

13. (income or GDP or GNP or PPP).mp.

14. (health care or health care access or health care utili*).mp.

15. 4 or 5 or 6 or 7 or 8 or 9 or 10 or 11 or 12 or 13 or 14

16. (village or district or county or town or city or countr* or urban* or rural or provinc* or municipal* or nation*).mp.

17. (SARS or SARS virus or COVID-19 or wuhan virus or 2019-nCOV or coronavirus or Middle East Respiratory Syndrome Coronavirus or ebola or H1N1 or flu or influenza or communicable).mp.

18. exp communicable disease/

19. 17 or 18

20. 3 and 15 and 16 and 19

**China National Knowledge Infrastructure (searched in simplified Chinese)**

Keyword(“quarantine, event of lockdown”) AND Keyword(“SARS” OR “COVID” OR “coronavirus” OR “H1N1” OR “Ebola” OR “MERS”)

**WHO COVID-19 database:**

TW:"Social" or "societal" or "lockdown" or "quarantine" or "mass quarantine"**Supplement 2. List of variables in the coding framework**

1. Title of article

2. Author names

3. Year of publication

4. Journal

5. Country

6. Language

7. Abstract

8. Setting

9. Target disease

10. Type of study (e.g. observational/analytical)

11. Study duration

12. Intervention element

13. Intervention implementer

14. Target population

15. Name of funding agency

16. Results reported in other reviews (e.g. Lancet systematic review - Brooks et al. 2020)

17. Study sample size

18. Year data collected

19. Study population

20. Study design

21. Main outcome variables

22. Other outcome variables

23. Main predictors

24. Reviewer's comments (Was its study design robust? Why/Why not?)

25. Themes identified from the Results/Findings section of selected papers:

a. Economic: macro/micro economic themes

b. Psychological

c. Job/employment

d. Health care access

e. School/education

f. Travel

g. Food/resources

h. Others

26. Themes identified from the Discussion section of selected papers:

a. Economic: macro/micro economic themes

b. Psychological

c. Job/employment

d. Health care access

e. School/education

f. Travel

g. Food/resources

h. Others

27. Overall summary of the included paper

28. Recommendation(s) on how to tackle social consequences

29. Reviewer's comments (Reliability and trustworthiness on the findings. Why/Why not?)

30. Theory/rationale of effective quarantine (What theory?)

31. Methods applied for data collection

a. Focus group discussion, medical records, interviews, surveys (tick all that apply)

b. Cross-sectional or longitudinal study

c. Sampling strategy: whole sampling, random, representative, purposive, convenience

**Supplement 3. Additional references listed in Table 2**

1. Infection Prevention and Control Canada. Pandemic (H1N1) 2009 virus. 2014. https://ipac-canada.org/pandemic-h1n1-resources.php (accessed May 31, 2020).
2. Public Health Agency of Canada. Public health guidance for the prevention and management of Influenza-like-illness (ILI), including the pandemic (H1N1) 2009 influenza virus related to mass gatherings. 2009. https://web.archive.org/web/20090916125514/http://www.phac-aspc.gc.ca/alert-alerte/h1n1/phg-ldp-eng.php (accessed May 31, 2020) (archived).
3. Active Learning Network for Accountability and Performance in Humanitarian Action (ALNAP). Monrovia city corporation’s slum initiative: preparation of Monrovia slum upgrading initiative. 2008. https://www.alnap.org/system/files/content/resource/files/main/monrovia-slum.pdf (accessed May 31, 2020).
4. The World Bank Group. Countries and economies. 2020. https://data.worldbank.org/country (accessed May 31, 2020).
5. Health Canada. Learning from SARS: renewal of public health in Canada. 2004. https://www.canada.ca/content/dam/phac-aspc/migration/phac-aspc/publicat/sars-sras/pdf/sars-e.pdf (accessed May 31, 2020).
6. Pang X, Zhu Z, Xu F, Guo J, Gong X, Liu D, et al. Evaluation of control measures implemented in the severe acute respiratory syndrome outbreak in Beijing, 2003. *JAMA* 2003; 290:3215–21.

## Supplement 4. Fifteen full-text articles assessed in the systematic review

1 Abramowitz SA, McLean KE, McKune SL, et al. Community-centered responses to Ebola in urban Liberia: the view from below. PLoS Negl Trop Dis 2015; 9:e0003706.

2 Blendon RJ, Benson JM, DesRoches CM, et al. The public’s response to severe acute respiratory syndrome in Toronto and the United States. Clin Infect Dis 2004; 38:925–931.

3 Cava MA, Fay KE, Beanlands HJ, et al. The experience of quarantine for individuals affected by SARS in Toronto. Public Health Nurs 2005; 22:398–406.

4 Charania NA, Tsuji LJ. Assessing the effectiveness and feasibility of implementing mitigation measures for an influenza pandemic in remote and isolated First Nations communities: a qualitative community-based participatory research approach. Rural Remote Health 2013; 13:2566.

5 DiGiovanni C, Conley J, Chiu D, Zaborski J. Factors influencing compliance with quarantine in Toronto during the 2003 SARS outbreak. Biosecurity Bioterrorism Biodefense Strateg Pract Sci 2004; 2:265–272.

6 Gostin LO, Bayer R, Fairchild AL. Ethical and legal challenges posed by severe acute respiratory syndrome. JAMA 2003; 290:3229–3237.

7 Hawryluck L, Gold WL, Robinson S, et al. SARS control and psychological effects of quarantine, Toronto, Canada. Emerg Infect Dis 2004; 10:1206–1212.

8 John N, Casey S, Carino G, McGovern T. Lessons never learned: crisis and gender-based violence. Dev World Bioeth 2020; 00:1–4.

9 Kodish SR, Bio F, Oemcke R, et al. A qualitative study to understand how Ebola Virus Disease affected nutrition in Sierra Leone – a food value-chain framework for improving future response strategies. PLoS Negl Trop Dis 2019; 13:e0007645.

10 Mihashi M, Otsubo Y, Yinjuan X, et al. Predictive factors of psychological disorder development during recovery following SARS outbreak. Health Psychol 2009; 28:91–100.

11 Pellecchia U, Crestani R, Decroo T, et al. Social consequences of Ebola containment measures in Liberia. PLoS One 2015; 10:e0143036.

12 Reynolds DL, Garay JR, Deamond SL, et al. Understanding, compliance and psychological impact of the SARS quarantine experience. Epidemiol Infect 2008; 136:997–1007.

13 Tracy CS, Rea E, Upshur RE. Public perceptions of quarantine: community-based telephone survey following an infectious disease outbreak. BMC Public Health 2009; 9:470.

14 Wilken JA, Pordell P, Goode B, et al. Knowledge, attitudes, and practices among members of households actively monitored or quarantined to prevent transmission of Ebola Virus Disease – Margibi county, Liberia: February–March 2015. Prehosp Disaster Med 2017; 32:673–678.

15 Zhang SX, Wang Y, Rauch A, Wei F. Unprecedented disruption of lives and work: health, distress and life satisfaction of working adults in China one month into the COVID-19 outbreak. Psychiatry Res 2020; 288:112958

**Supplement 5. PRISMA Checklist**

| **Section/topic** | **#** | **Checklist item** | | **Reported on page #** |
| --- | --- | --- | --- | --- |
| **TITLE** | | | |  |
| Title | 1 | Identify the report as a systematic review, meta-analysis, or both. | | p. 1 |
| **ABSTRACT** | | | |  |
| Structured summary | 2 | Provide a structured summary including, as applicable: background; objectives; data sources; study eligibility criteria, participants, and interventions; study appraisal and synthesis methods; results; limitations; conclusions and implications of key findings; systematic review registration number. | | p. 3-4 |
| **INTRODUCTION** | | | |  |
| Rationale | 3 | Describe the rationale for the review in the context of what is already known. | | p. 5 |
| Objectives | 4 | Provide an explicit statement of questions being addressed with reference to participants, interventions, comparisons, outcomes, and study design (PICOS). | | p. 6 |
| **METHODS** | | | |  |
| Protocol and registration | 5 | Indicate if a review protocol exists, if and where it can be accessed (e.g., Web address), and, if available, provide registration information including registration number. | | p. 7 |
| Eligibility criteria | 6 | Specify study characteristics (e.g., PICOS, length of follow-up) and report characteristics (e.g., years considered, language, publication status) used as criteria for eligibility, giving rationale. | | p. 7 |
| Information sources | 7 | Describe all information sources (e.g., databases with dates of coverage, contact with study authors to identify additional studies) in the search and date last searched. | | p. 7 |
| Search | 8 | Present full electronic search strategy for at least one database, including any limits used, such that it could be repeated. | | p. 7 |
| Study selection | 9 | State the process for selecting studies (i.e., screening, eligibility, included in systematic review, and, if applicable, included in the meta-analysis). | | p. 7 |
| Data collection process | 10 | Describe method of data extraction from reports (e.g., piloted forms, independently, in duplicate) and any processes for obtaining and confirming data from investigators. | | p. 8 |
| Data items | 11 | List and define all variables for which data were sought (e.g., PICOS, funding sources) and any assumptions and simplifications made. | | p. 7 |
| Risk of bias in individual studies | 12 | Describe methods used for assessing risk of bias of individual studies (including specification of whether this was done at the study or outcome level), and how this information is to be used in any data synthesis. | | p. 8 |
| Summary measures | 13 | State the principal summary measures (e.g., risk ratio, difference in means). | | p. 7 |
| Synthesis of results | 14 | Describe the methods of handling data and combining results of studies, if done, including measures of consistency (e.g., I^2^) for each meta-analysis. | | p. 7 |
| Risk of bias across studies | 15 | Specify any assessment of risk of bias that may affect the cumulative evidence (e.g., publication bias, selective reporting within studies). | | N/A |
| Additional analyses | 16 | Describe methods of additional analyses (e.g., sensitivity or subgroup analyses, meta-regression), if done, indicating which were pre-specified. | | N/A |
| **RESULTS** | | |  | |
| Study selection | 17 | Give numbers of studies screened, assessed for eligibility, and included in the review, with reasons for exclusions at each stage, ideally with a flow diagram. | p. 13 | |
| Study characteristics | 18 | For each study, present characteristics for which data were extracted (e.g., study size, PICOS, follow-up period) and provide the citations. | p. 9-11 | |
| Risk of bias within studies | 19 | Present data on risk of bias of each study and, if available, any outcome level assessment (see item 12). | p. 20 | |
| Results of individual studies | 20 | For all outcomes considered (benefits or harms), present, for each study: (a) simple summary data for each intervention group (b) effect estimates and confidence intervals, ideally with a forest plot. | p. 9-11 | |
| Synthesis of results | 21 | Present results of each meta-analysis done, including confidence intervals and measures of consistency. | N/A | |
| Risk of bias across studies | 22 | Present results of any assessment of risk of bias across studies (see Item 15). | N/A | |
| Additional analysis | 23 | Give results of additional analyses, if done (e.g., sensitivity or subgroup analyses, meta-regression [see Item 16]). | N/A | |
| **DISCUSSION** | | |  | |
| Summary of evidence | 24 | Summarize the main findings including the strength of evidence for each main outcome; consider their relevance to key groups (e.g., healthcare providers, users, and policy makers). | p. 24 | |
| Limitations | 25 | Discuss limitations at study and outcome level (e.g., risk of bias), and at review-level (e.g., incomplete retrieval of identified research, reporting bias). | p. 27-29 | |
| Conclusions | 26 | Provide a general interpretation of the results in the context of other evidence, and implications for future research. | p. 29 | |
| **FUNDING** | | |  | |
| Funding | 27 | Describe sources of funding for the systematic review and other support (e.g., supply of data); role of funders for the systematic review. | p. 2 | |

Adapted from: Moher D, Liberati A, Tetzlaff J, Altman DG, The PRISMA Group (2009). Preferred Reporting Items for Systematic Reviews and Meta-Analyses: The PRISMA Statement. PLoS Med 6(7): e1000097. doi:10.1371/journal.pmed1000097
